# Structure Function Revisited: A Simple Tool for Complex Analysis of Neuronal Activity

**DOI:** 10.3389/fnhum.2017.00409

**Published:** 2017-08-14

**Authors:** Federico Nanni, Daniela S. Andres

**Affiliations:** Science and Technology School, National University of San Martin (UNSAM) San Martin, Argentina

**Keywords:** Parkinson's disease, neuronal activity, interspike intervals, 6-hydroxydopamine, alertness, basal ganglia, complexity, temporal structure

## Abstract

Neural systems are characterized by their complex dynamics, reflected on signals produced by neurons and neuronal ensembles. This complexity exhibits specific features in health, disease and in different states of consciousness, and can be considered a hallmark of certain neurologic and neuropsychiatric conditions. To measure complexity from neurophysiologic signals, a number of different nonlinear tools of analysis are available. However, not all of these tools are easy to implement, or able to handle clinical data, often obtained in less than ideal conditions in comparison to laboratory or simulated data. Recently, the temporal structure function emerged as a powerful tool for the analysis of complex properties of neuronal activity. The temporal structure function is efficient computationally and it can be robustly estimated from short signals. However, the application of this tool to neuronal data is relatively new, making the interpretation of results difficult. In this methods paper we describe a step by step algorithm for the calculation and characterization of the structure function. We apply this algorithm to oscillatory, random and complex toy signals, and test the effect of added noise. We show that: (1) the mean slope of the structure function is zero in the case of random signals; (2) oscillations are reflected on the shape of the structure function, but they don't modify the mean slope if complex correlations are absent; (3) nonlinear systems produce structure functions with nonzero slope up to a critical point, where the function turns into a plateau. Two characteristic numbers can be extracted to quantify the behavior of the structure function in the case of nonlinear systems: (1). the point where the plateau starts (the inflection point, where the slope change occurs), and (2). the height of the plateau. While the inflection point is related to the scale where correlations weaken, the height of the plateau is related to the noise present in the signal. To exemplify our method we calculate structure functions of neuronal recordings from the basal ganglia of parkinsonian and healthy rats, and draw guidelines for their interpretation in light of the results obtained from our toy signals.

## Introduction

The nervous system is complex at many levels. It is built as a network of nonlinear elements with complex dynamics themselves: the neurons (Rulkov, 2002; Korn and Faure, 2003). As a result, the output of the nervous system exhibits complex dynamics at multiple scales, which is reflected in neural signals from the level of single neurons, to microcircuits and larger neuronal networks. This complexity can be measured from different kinds of data, for instance microelectrode recordings (MER), electroencephalograms (EEG) and functional magnetic resonance imaging (fMRI) (Mpitsos et al., 1988; Elger et al., 2000; Yang et al., 2013a,b). Although the theoretical concepts used to characterize these different signals are not scale specific (chaoticity, entropy, nonlinearity, fractality), the tools of analysis required to calculate complexity measures from different signals do differ, and need to be tuned for each particular case.

Our motivation comes from the observation of complex properties in the neuronal activity of the basal ganglia (Darbin et al., 2006; Lim et al., 2010; Andres et al., 2011). Different complexity measures of basal ganglia activity show a correlation to different physiologic/pathologic conditions, like arousal level or the presence of specific pathologies (dystonia, parkinsonism) (Sanghera et al., 2012; Andres et al., 2014a; Alam et al., 2015). Even more, some of these measures can be modified with therapeutic interventions, suggesting that a correct characterization of basal ganglia complexity has potentially high clinical impact (Dorval et al., 2008; Lafreniere-Roula et al., 2010). However, complexity measures are not always easily transferred to the clinic. Nonlinear tools are sensitive not only to parameters' settings, but also to the number of data analyzed (length of the recordings), noise and linear correlations present in the signals. Because of that, the correct implementation of nonlinear tools depends critically on the behavior of the tool at hand for the particular system under study (Schreiber, 1999).

In this context, the temporal structure function emerged as a powerful tool for the analysis of basal ganglia activity. In previous work we tested this tool on human, animal and simulated neuronal data, and observed a correlation of abnormalities of the temporal structure of basal ganglia spike trains with parkinsonism (Andres et al., 2014b, 2015, 2016). Importantly, the calculation of temporal structure functions of spike trains is robust and efficient computationally. However, the interpretation of results remains difficult, partly because the use of temporal structure functions for the characterization of spike trains is relatively new. Here, we perform an analysis of the temporal structure of toy signals, i.e., signals with known properties, as a means to characterize the behavior of the tool. This task has been partly attempted before, but the analysis did not include a study of any complex system or a comparison with neuronal data, and is therefore incomplete to our purpose (Yu et al., 2003). Our goal is to draw general guidelines for the interpretation of structure functions of neuronal data. To illustrate the method, we calculate the structure function of spike trains obtained from the basal ganglia of healthy and parkinsonian rats during the transition from deep anesthesia to alertness.

## The temporal structure function

### Definition

Consider the following time series:

(1) $$\begin{array}{r} I\left(t\right)=\left\{I_{t1},I_{t2},\ldots ,I_{tn}\right\} \end{array}$$

where *I*_*tn*_ are successive recordings of the variable *I* at the times *t*1, *t*2 to *tn*. The length of the time series is *n*. For this time series, the temporal structure function is a function of the scale τ and of order *q*, defined as the average of the absolute value of the differences between elements of the time series separated by time lags corresponding to the scale τ, elevated to the power of *q* (Lin and Hughson, 2001):

(2) $$\begin{array}{r} S_{q}\left(\tau \right)=\langle |\Delta I\left(\tau \right){|}^{q}\rangle \end{array}$$

Here, Δ denotes the difference, |·| denotes the absolute value and 〈·〉 denotes the average.

It is often the case for complex systems that structure functions of increasing order follow a relation like (Lin and Hughson, 2001):

(3) $$\begin{array}{r} S_{q}\left(\tau \right)\propto \tau^{\zeta \left(q\right)}. \end{array}$$

This justifies the plotting of structure functions with double logarithmic axes, since the exponent ζ can be recovered from this plot as the slope of the linear regression, because:

(4) $$\begin{array}{r} \log\left(S_{q}\left(\tau \right)\right)=\zeta_{q}\cdot \log\left(\tau \right). \end{array}$$

Therefore, when discussing the slope of *S*_*q*_(τ) plotted in log-log, we are in fact making reference to the exponent ζ. In addition, the double logarithmic plotting enhances the visualization of small scales.

An analogous definition applies to spatial series of data, where *I* (*x*) are recordings obtained simultaneously in a spatial arrangement, instead of at successive times (Stotskii et al., 1998). In that case, the structure function is called spatial structure function, in opposition to the temporal structure function, in which we are interested here. In the case of a velocity field, a linear transformation (i.e., the velocity) relates the spatial and the temporal structure functions.

### Step by step algorithm for the calculation of the temporal structure function

**Figure 1:**
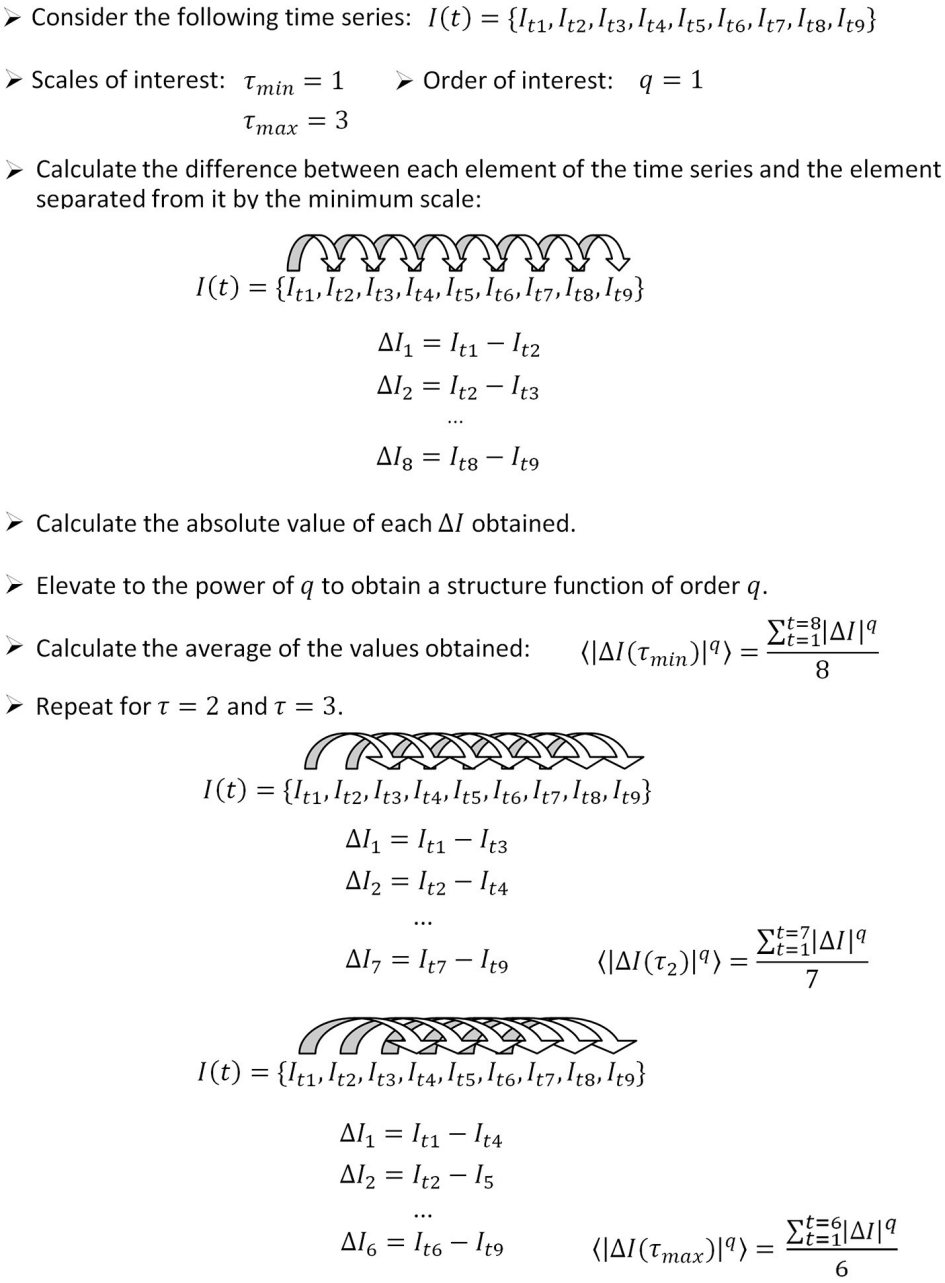
How to apply the algorithm for the calculation of the temporal structure function to a sample time series. In this example, we calculate the structure function of order 1, therefore the power step is not needed. The number of differences computed for each scale (used as the denominator to calculate the average) is equal to the length of the time series minus the scale. We show how to calculate three points of a temporal structure function (*S*(τ_1, 2, 3_)), corresponding to scales 1–3. Typically, large scale ranges are of interest to characterize short and long term dynamics (for instance *S*(τ_1−1000_)).

In this section, we introduce briefly a step by step algorithm for the calculation of the temporal structure function from a given signal. The procedure is illustrated in Figure 1. To calculate the temporal structure function of a signal *I* (*t*), proceed as follows.

Step 1: Define the range of scales of interest, going from the minimum scale τ_*min*_ to the maximum scale τ_*max*_.

Step 2: Define the range of orders of interest, going from the minimum order *q*_*min*_ to the maximum order *q*_*max*_.

Step 3: Calculate the difference between each element of the time series *I*_*t*_ and the element separated from it by a number of elements equal to τ_*min*_:

(5) $$\begin{array}{r} I_{t}-I_{t\;+\;\tau \text{\_}min}. \end{array}$$

The number of values obtained is equal to the length of the time series minus the scale (*n* − τ_*min*_).

Step 4: Calculate the absolute value of the differences obtained from step 3.

Step 5: Elevate the values obtained from step 4 to the power of *q*_*min*_.

Step 6: Calculate the average of all the values obtained from step 5.

Step 7: Increase τ and repeat steps 3–6 until τ_*max*_ is reached. In this way, one obtains *S*_*q*_*min*_(τ).

Step 8: To calculate structure functions of higher order, repeat steps 3–7 until *q*_*max*_ is reached.

In this paper we focus on temporal structure functions of order 1, in which case steps 5 and 8 are needless. The behavior of the slope of the structure function at increasing q indicates the kind of fractal structure present in the signal: mono- vs. multifractal properties (Lin and Hughson, 2001). These properties might indeed be useful for a better characterization of neuronal signals. However, in previous work we found that the structure function of pallidal neurons of the rat shows no great differences for orders up to *q* = 6 (Andres et al., 2014b). Therefore in this paper we chose to stay at order one, for simplicity. As is good practice, we normalized *S* (τ) by dividing by the initial value, and therefore all the structure functions shown in this paper start at *S* (1) = 1.

**Figure 2:**
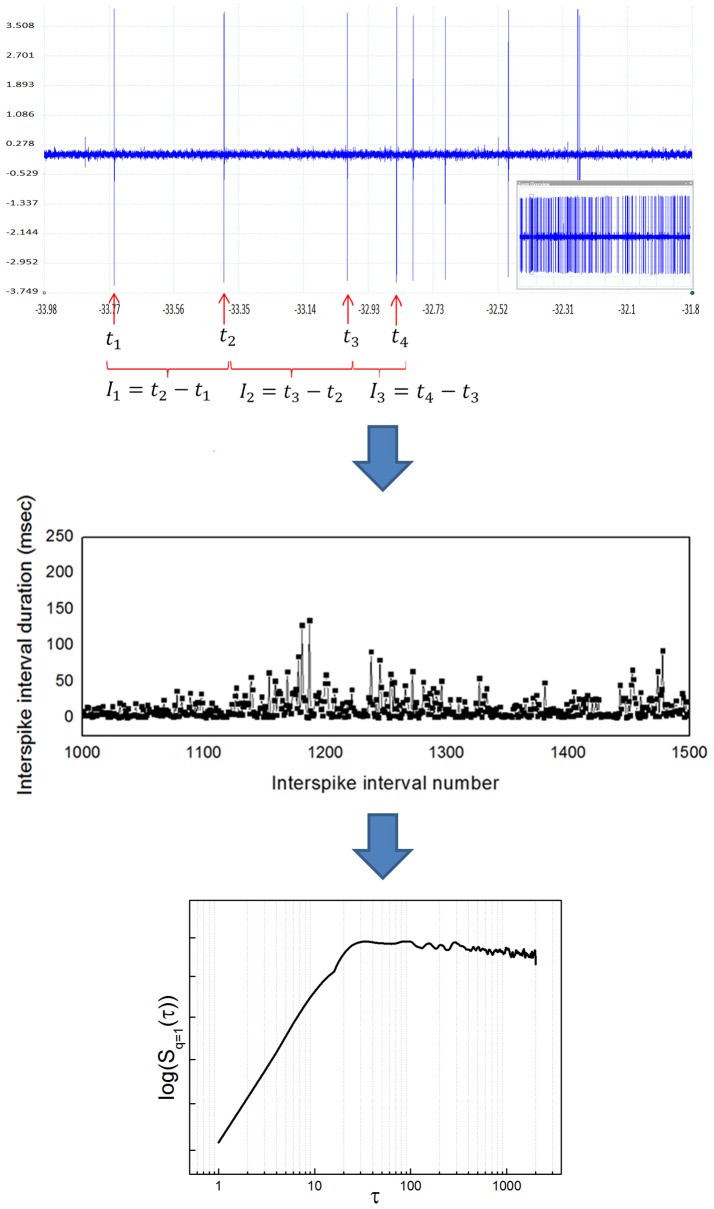
Transformation from a raw neuronal recording into a temporal structure function. **(Upper)** Sample raw extracellular microelectrode recording of neuronal activity. This recording was obtained from the entopeduncular nucleus of a healthy rat (technical details can be found in Andres et al., 2014a). The vertical axis indicates electric potential (mV) and the horizontal axis indicates time (s). The inlet at the lower right shows the whole recording, from which a zoom is shown in the bigger window. Individual spikes are marked with a red arrow. Once spikes are classified as belonging to a single neuron's activity, interspike intervals (ISI) are calculated as shown (ISI = time elapsed between the occurrence of a spike and the next). **(Middle)** Sample time series of interspike intervals, obtained from a neuronal recording like the one shown in the upper panel. The vertical axis indicates ISI duration (ms) and the horizontal axis indicates ISI number (position in the time series). Notice the high variability of the ISI, typical of complex systems. **(Lower)** Temporal structure function obtained from a time series of ISI like the one shown in the middle panel. The vertical axis is the value of the function *S*(τ) and the horizontal axis is the scale τ. In pallidal neurons it is common to observe a positive slope of the function at lower scales, followed by a breakpoint and a plateau at higher scales, also typical of complex systems. The double logarithmic scale helps visualization of smaller τ.

If the signal of interest is a neuronal recording of spiking activity, some previous conditioning is needed before applying the algorithm described. Previous steps include: (1) isolate single neuronal activity employing some spike sorting algorithm (see for example Quiroga et al., 2004), (2) detect the times of occurrence of spikes, and (3) build a time series of interspike intervals (ISI) to obtain the time series *I* (*t*). Figure 2 illustrates the whole transformation process from the raw neuronal recording to the structure function.

## Temporal structure of toy systems

### Random time series

**Figure 3:**
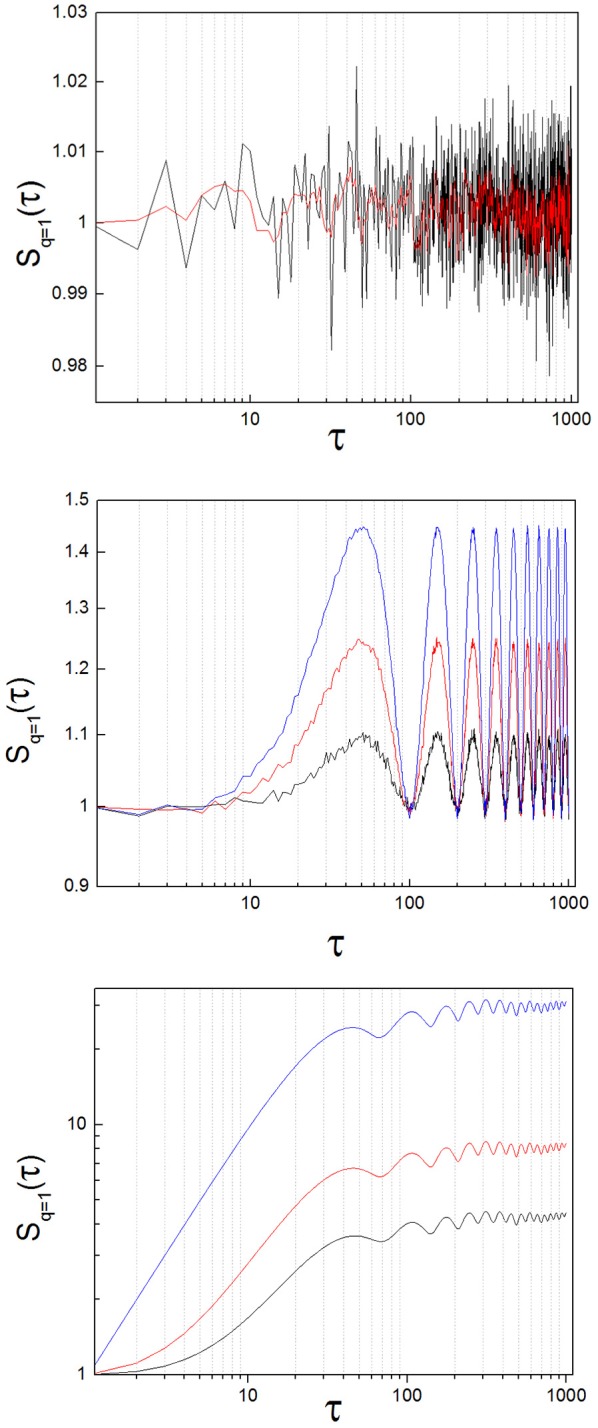
Structure function of toy signals, with and without added noise. **(Upper)** Random signal, before (black) and after (red) applying a low-pass filter. The slope calculated with a linear regression lies around zero for both cases. **(Middle)** Oscillatory signal (sin(x)), with increasing levels of added noise, (blue: x1.0, red: x1.5, black: x2.0; see the text). The slope is zero on average, but the oscillations of the signal are clearly translated into the structure function. As added noise increases, the amplitude of the oscillations diminishes. **(Lower)** Lorenz system (x variable; parameters: σ = 10, ρ = 28, β = 8/3), with increasing levels of added noise, (blue: no noise, red: x1.0, black: x2.0; see the text). The breaking point lies around 40 < τ_1_ < 110 for this example. Observe that the position of the breaking point in the structure function does not change as added noise increases, but the height of the plateau diminishes. In the limit, the initial ascending phase disappears and the slope of the structure function is zero at all scales, as random dynamics prevail over the nonlinear system.

Theoretical work demonstrates that the structure function of random signals has mean slope equal zero (Lin and Hughson, 2001). To test the practical implementation of this statement, we generated 30 random signals that followed a normal distribution (mean = 1, *SD* = 0.1) with a length of 10^4^ numbers. We obtained *S*(τ) of these random signals and calculated the slope of *S*(τ) with a linear regression (Figure 3, upper panel). The slope had a value close to zero for all cases (8.07·10^−8^ ± 2.31·10^−7^: mean ± standard deviation; *SD*).

A well-known measure in time series analysis is the autocorrelation function. In the case of random series, the autocorrelation falls rapidly to zero indicating independent behavior of the elements of a signal. This behavior is hardly differentiated from the rapid loss of autocorrelation of complex nonlinear systems with highly variable output, like the Lorenz system. On the contrary, the zero-slope behavior of the structure function of random signals is clearly different from the temporal structure of complex systems.

### Oscillatory signals

We calculated *S*(τ) from *sin*(*x*) (Figure 3, middle panel). Structure functions of oscillatory signals oscillate steadily between a lower and an upper bound. In general, the frequency of oscillation is translated into the structure function following the rule

(6) $$\begin{array}{r} \omega \left(\tau \right)=\omega \left(I\left(t\right)\right)/samp, \end{array}$$

where *I* (*t*) is the original signal and *samp* is the sampling rate. As is the case with random signals, the mean slope of *S*(τ) of perfectly oscillatory signals is close to zero (linear regression, slope = −1.18 x 10^−4^).

To test the effect of adding increasing noise levels to an oscillatory signal, we multiplied a time series of random numbers with normal distribution (0 ± 1, mean ± *SD*) by a factor of 1.0, 1.5 and 2.0 successively, and added it to *sin* (*x*). We calculated *S*(τ) from the three resulting time series. The results of adding increasing noise levels are plotted in blue, red and black in the middle panel of Figure 3. The slope of the temporal structure function *S*(τ) remains around zero as the noise level is increased, while its amplitude diminishes, due to the effect of the random variable on the average term of the structure function. It needs to be noted that the slope of *S*(τ) of periodic signals is not zero, if scales smaller than the period are considered. However, the mean slope of *S*(τ) of periodic oscillatory signals is close to zero, if a time sufficiently longer than the period itself is measured, which is observed in our results.

### Nonlinear systems

To analyze the structure function of nonlinear systems with complex properties we obtained a signal representative of the time evolution of the well-known, chaotic Lorenz attractor (Strogatz, 1994). We integrated numerically the Lorenz equations with the Euler method, then extracted the temporal variable *x*(*t*) and finally calculated the structure function of *x*(*t*) (Figure 3, lower panel). The results show a structure function with a positive slope at small scales and a clear breaking point. At this breaking or inflection point, which we have called τ_1_ in previous work, the function turns into a plateau, turning more or less abruptly into a zero-sloped function (Andres et al., 2015). This behavior is related to the loss of autocorrelation of the system, associated to its chaoticity. However, the autocorrelation function *C*(τ) is very similar for random and complex systems. On the contrary, complex systems with long range, nonlinear correlations (like the Lorenz system) exhibit structure functions *S*(τ) dramatically different from random and oscillatory signals. The main difference is observed in the mean slope of *S*(τ), which is no longer zero at every scale.

To test the effect of added noise on this nonlinear system, we followed a similar procedure as with the oscillatory signals. We multiplied a time series of random numbers with normal distribution (0 ± 1, mean ± *SD*) by a factor of 1.0 and 2.0 successively, and added it to the nonlinear time series. The position of the breaking point in the function does not change as noise is added to the signal (Figure 3, lower panel, blue line: no noise, red and black lines: increasing noise levels). However, the height of the plateau of the structure function is sensitive to the noise level, and as a consequence the slope of the function tends to zero as noise is added.

## Neuronal recordings

**Figure 4:**
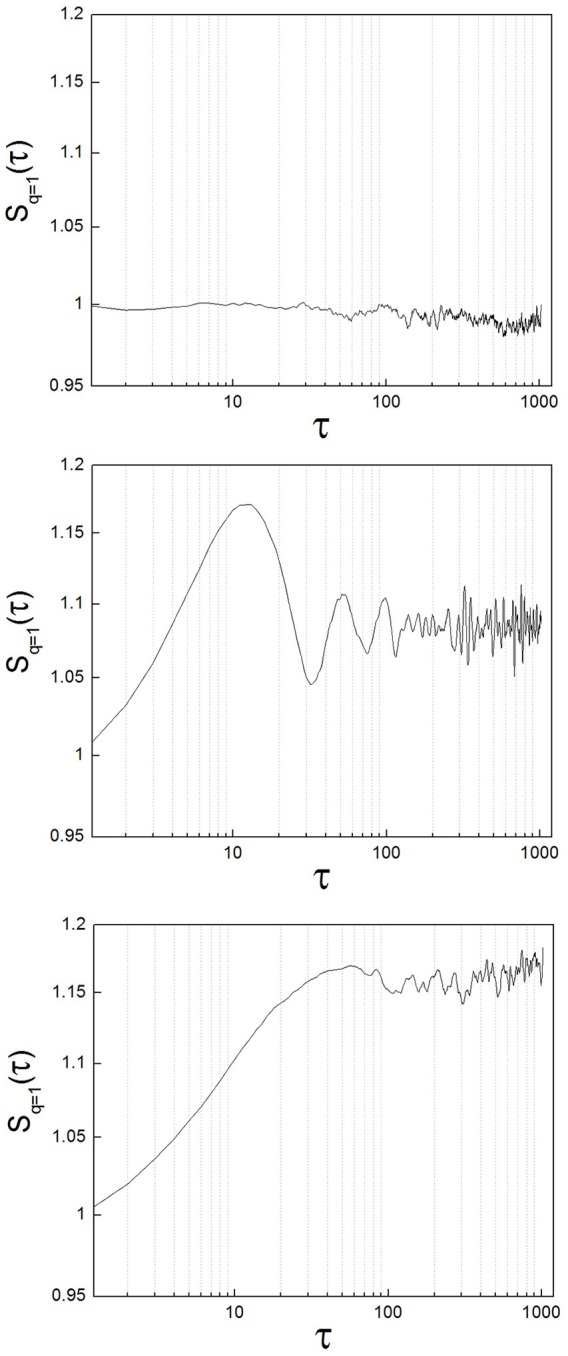
Different types of structure functions from neuronal recordings show features of random, oscillatory, and nonlinear systems. **(Upper)** Sample neuron with a structure function showing zero-slope at all scales, indicating random behavior. **(Middle)** Sample neuron with a structure function showing oscillations. **(Lower)** This case is the most representative of all the neurons analyzed (64%). The structure function has clear, nonlinear behavior.

To exemplify the implementation of the method, we analyzed neuronal recordings of the entopeduncular nucleus of the rat (analogous to the internal segment of the globus pallidus in the primate/human: GPi). The experimental protocol was revised and approved by FLENI Ethics Committee, Buenos Aires, Argentina. Recordings belonged to two groups of animals: healthy and parkinsonian rats. Detailed methodological information can be found in Andres et al. (2014a). Briefly, in adult Sprague-Dawley rats we induced Parkinsonism implementing the 6-hydroxydopamine (6-OHDA) partial retrograde lesion of the nigrostriatal pathway. We recorded spontaneous neuronal activity of the GPi under intraperitoneal anesthesia with chloral-hydrate and at increasing levels of alertness. Alertness levels are as described in Andres et al. (2014a): level (1) deep anesthesia; level (2) mild alertness; level (3) full alertness. We analyzed a total of 45 neuronal recordings, belonging to the following groups: from 11 healthy animals, 22 neuronal recordings (level 1: *n* = 5; level 2: *n* = 11; level 3: *n* = 6) and from 9 parkinsonian animals, 23 neuronal recordings (level 1: *n* = 7; level 2: *n* = 9; level 3: *n* = 7). In Figure 4 we show sample structure functions of neuronal recordings, to illustrate the occurrence in neuronal activity of features such as those of toy systems (randomness, oscillations and nonlinear properties). A majority of the recordings (64%) showed marked nonlinear behavior (corresponding to type A neurons of Andres et al., 2015). Additionally, 13% of the recordings presented clear oscillations. A minority of neurons (33%) presented a zero-slope of the structure function at all scales, indicating random behavior. These percentages did not vary significantly between the control and the parkinsonian group or at different levels of alertness, but these results need to be further tested with greater numbers of recordings.

**Figure 5:**
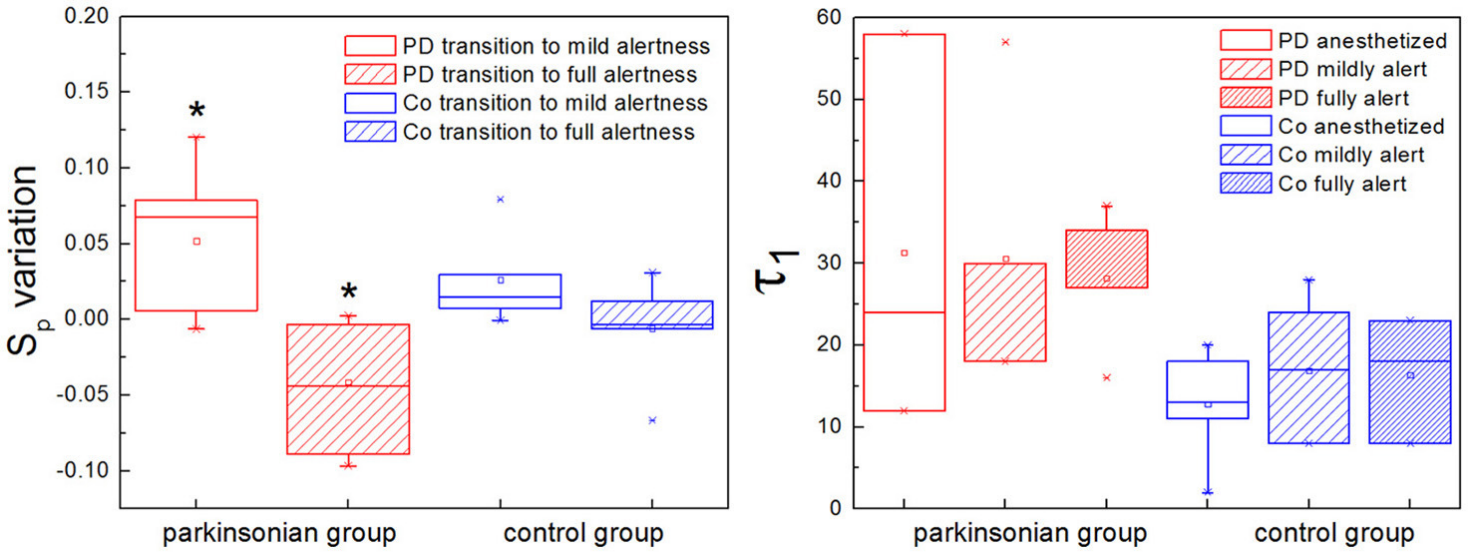
The plateau height (*S*_*p*_) and the inflection point (τ_1_) characterize the structure function if nonlinearity is present. **(Left)** The plateau height *S*_*p*_ can be used to compare the activity of the same neuron at different time points, assuming that recording conditions do not change. In the parkinsonian group, *S*_*p*_ varies significantly more between anesthesia and mild alertness than between mild and full alertness (^*^p < 0.01). This is new evidence showing that in Parkinson's disease (PD) basal ganglia neurons are unable to handle the awakening process well. This effect is not observed in the control group of animals (Co). **(Right)** The inflection point τ_1_ is higher in the parkinsonian group for all alertness levels, with a more pronounced effect as alertness increases. These results are not statistically significant (*p* > 0.05), and need to be confirmed with larger experimental data.

Two characteristic numbers can be extracted from *S*(τ) to quantify its behavior, when a change of slope typical of nonlinear systems is observed: the inflection point τ_1_, where the slope of the function changes, and the height of the plateau, which we call *S*_*p*_. In previous work we developed an algorithm for the calculation of τ_1_, and observed a higher τ_1_ in GPi neurons with nigrostriatal lesion (Andres et al., 2015). This observation was done under conditions of full alertness, i.e., animals were under local anesthesia plus analgesia, alert and head restrained at the moment of the surgery. Now we calculated τ_1_ applying the same algorithm from neuronal recordings obtained during the whole arousal process, going from deep anesthesia, to mild and full alertness. All statistical comparisons were calculated applying the Kolmogorov-Smirnov test; results were considered statistically significant when *p* < 0.05. Results show that τ_1_ is higher in the parkinsonian group at all alertness levels, with a more pronounced effect as alertness increases (i.e., as the animal awakens from anesthesia; Figure 5, right panel). These results were not statistically significant (*p* > 0.05) and need to be tested on more experimental data.

We calculated the plateau height *S*_*p*_ as the mean value of *S*(τ) for 100 < τ < 200, a range where all the neurons analyzed had reached a plateau, if this was present. We have shown in the previous section that the plateau height is sensitive to the amount of noise added to a signal. In this sense *S*_*p*_ might not be reliable as a raw measure to compare neuronal data corresponding to different experimental groups. This disadvantage can be overcome by studying variations of *S*_*p*_ instead of raw values, i.e., subtracting a given *S*_*p*_ from a previous value of itself obtained under the same recording conditions. In our study case we recorded activity from single neurons during long periods of time (1–3 h), and we can safely assume that environmental conditions (electrical noise and any other source of interference) did not vary during the whole recording. We calculated *S*_*p*_ from isolated segments of activity obtained at the beginning, middle and end of the recording, corresponding to deep anesthesia, mild alertness and full alertness, respectively. Thus, we obtained the following values of *S*_*p*_: *S*_*p*1−2_, as the difference between the plateau height at mild alertness minus the plateau height at deep anesthesia, and *S*_*p*2−3_, as the difference between the plateau height at full alertness minus the plateau height at mild alertness. In the control group we did not observe any difference between *S*_*p*1−2_ and *S*_*p*2−3_, whereas under parkinsonian conditions *S*_*p*1−2_ was significantly higher than *S*_*p*2−3_ (*p* < 0.01; Figure 5, left panel).

## Summary and conclusion: guidelines for the interpretation of the temporal structure *S*(τ) of neuronal signals

Nonlinear properties of neuronal activity are critical for normal basal ganglia functioning and deteriorate in specific ways in disease, in particular in movement disorders (Parkinson's disease, dystonia, and others) (Montgomery, 2007; Darbin et al., 2013; Alam et al., 2015). Even more, therapeutic interventions are able to restitute such properties to normal, suggesting the clinical importance of quantifying nonlinear features of neuronal activity (Rubin and Terman, 2004; Lafreniere-Roula et al., 2010). However, up to now the community has not agreed on any method as a gold standard to quantify nonlinear properties of the basal ganglia. This is partly due to difficulties in the implementation of nonlinear methods of analysis, which are typically sensitive to a wide range of parameters. Opposed to that, the temporal structure function *S*(τ) is a nonlinear tool of analysis easy to implement, and robust to short recordings, but it is not well known and therefore difficult to interpret. We analyzed the behavior of *S*(τ) from signals with known properties (toy systems), and observed that: (1) *S*(τ) has zero-slope at every scale for random systems; (2) *S*(τ) is oscillatory and bounded for oscillatory systems, and the frequency of oscillations can be recovered from *S*(τ) if the sampling rate is known; and (3) *S*(τ) has a positive slope at small scales for nonlinear systems, which changes to a plateau with zero-slope at large scales.

In the light of our observations for toy systems we analyzed a number of neuronal recordings of healthy and parkinsonian basal ganglia at different levels of alertness (from deep anesthesia to full alertness). In a majority of the neurons studied, nonlinear behavior was clearly present. In these cases we extracted two characteristic numbers from *S*(τ) to quantify its behavior: (1) the height of the plateau (*S*_*p*_), and (2) the scale of the inflection point or slope change (τ_1_). Since the plateau height is sensitive to the noise added to the signal, we used it in a relative way, measuring the changes of *S*_*p*_ for alertness transitions within single neuronal recordings, when we can assume that recording conditions were stationary. For parkinsonian neurons the change of plateau height from deep anesthesia to mild alertness was significantly higher than from mild to full alertness (*S*_*p*1−2_ > *S*_*p*2−3_, *p* < 0.01). This difference in the variation of *S*_*p*_ was not observed in the control group. The fact that *S*_*p*1−2_ is significantly higher than *S*_*p*2−3_ in parkinsonian animals is in agreement with previous observations indicating that the basal ganglia of animals with dopamine depletion do not handle well the awakening process (Andres et al., 2014a). Importantly, as a consequence of the averaging process *S*_*p*_ is independent from the frequency of discharge of the neurons by definition, solving a previous controversy about the structure function method (Darbin et al., 2016). Regarding the inflection point τ_1_, it was higher in Parkinson's disease than in control neurons with a more pronounced effect at higher alertness levels, but this effect was not statistically significant (*p* > 0.05) and needs to be further tested in larger studies. Although preliminary, our findings are relevant for understanding results obtained from human surgery on Parkinson's disease, usually performed with the patient awake, under local anesthesia only. We report on the inability of pallidal neurons with Parkinson's disease to handle normally the transition from anesthesia to alertness, which might be a key finding to better understand the pathophysiology of the basal ganglia.

In previous work, we determined that the positive slope at small scales of the log-log temporal structure function is associated with particular properties of neuronal dynamics. Specifically, in a neuronal network with nonlinear properties we showed that the slope depends on the coupling strength (Andres et al., 2014b). This indicates that the temporal structure function captures critical properties of the underlying dynamics of the system. Importantly, in our previous modeling study we observed that a smaller percentage of neurons behave in random fashion, which seems to be related to the stability of the system (Andres et al., 2014b). This finding is now reproduced in our experimental results.

Finally, we would like to draw some attention to other nonlinear tools that are available for the characterization of neurophysiologic signals (Amigó et al., 2004; Pereda et al., 2005; Song et al., 2007). Every tool shows advantages and shortcomings, making them more or less suitable for the study of specific neurologic systems. Recently Zunino et al. introduced two methods that seem to be particularly powerful for the analysis of physiologic time series (Zunino et al., 2015, 2017). A detailed review and comparison of the performance of the temporal structure function with these other tools is beyond the scope of this paper. Nevertheless, we are still interested in a detailed analysis of the temporal structure function, because it has previously shown to be useful for the characterization of neuronal spike trains obtained from human patients with Parkinson's disease, which tend to be short (around 5,000 data per time series) (Andres et al., 2016). Another limitation of our work is that we have studied the temporal structure function of only one nonlinear system (i.e., the Lorenz attractor). However, our results are supported by results from other well-known nonlinear systems, which exhibit similar behavior (Lin and Hughson, 2001).

To conclude, we would like to draw some brief guidelines for the interpretation of the temporal structure function of neuronal activity. The most important feature distinguishing *S*(τ) of random vs. complex signals is its slope. If *S*(τ) has zero slope for every τ, randomness can be assumed at the scales analyzed, meaning that the order of the events in the time series (in this case interspike intervals) is not different from what would be observed for an independent variable. On the other hand, if *S*(τ) has a first segment with positive slope then turning into a plateau, the behavior falls in the category of complex systems. In this case, one can look at τ_1_ and *S*_*p*_. The breaking point τ_1_ is related to the memory limit of the system, and therefore to its chaoticity. For scales below τ_1_ the order of events (ISI) is not random, and therefore memory or temporal organization is present. In other words, at scales smaller than τ_1_ the times of occurrence of single spikes are not independent from each other, but nonlinear organization plays a role in the signal. At scales larger than τ_1_ the system behaves in random fashion. Regarding the neural code, it can be assured that at scales larger than τ_1_ only a rate code or other averaged coding scheme can be used, since complex time patterns cannot be transmitted from neuron to neuron beyond the memory limit of the system (Bialek et al., 1991; Ferster and Spruston, 1995). The second quantitative measure that can be obtained from the temporal structure function is *S*_*p*_. While τ_1_ is related to the memory limit and is robust to noisy signals, *S*_*p*_ is sensitive to added noise. Therefore it is necessary to be cautious when comparing *S*_*p*_ between experimental data, if it cannot be assured that the data were obtained under similar conditions, in particular regarding external noise and interference. If recording conditions can be safely assumed to be stationary, then variations of *S*_*p*_ indicate a change in the power of the random components of the system. Finally, oscillations of the original signal are translated into the structure function, and the original frequency can be recovered from *S*(τ) if the sampling rate is known.

## Ethics statement

All animal experiments and procedures were conducted with adherence to the norms of the Basel Declaration. The experimental protocol was revised and approved by our local ethics committee CEIB, BuenosAires, Argentina.

## Author contributions

DA designed the study and conducted the experiments. FN processed and analyzed the data. Both authors contributed to the preparation of the manuscript and approved its final version.

### Conflict of interest statement

The authors declare that the research was conducted in the absence of any commercial or financial relationships that could be construed as a potential conflict of interest.
